# Risk Factors Associated with the Choice to Drink Bottled Water and Tap Water in Rural Saskatchewan

**DOI:** 10.3390/ijerph110201626

**Published:** 2014-01-29

**Authors:** Lianne McLeod, Lalita Bharadwaj, Cheryl Waldner

**Affiliations:** 1 Large Animal Clinical Sciences, University of Saskatchewan, 52 Campus Dr., Saskatoon SK S7N 5B4, Canada; E-Mail: cheryl.waldner@usask.ca; 2 School of Public Health, University of Saskatchewan, 107 Wiggins Road, Saskatoon SK S7N 5E5, Canada; E-Mail: lalita.bharadwaj@usask.ca

**Keywords:** drinking water, bottled water, tap water, water treatment, water quality, risk perception, aesthetic characteristics

## Abstract

A cross-sectional study investigated risk factors associated with choices to drink bottled water and tap water in rural Saskatchewan. Of 7,500 anonymous postal questionnaires mailed out, 2,065 responses were analyzed using generalized linear mixed models. Those who reported a water advisory (*p* < 0.001) or living in the area for ≤10 years (*p* = 0.01) were more likely to choose bottled water. Those who reported tap water was not safe to drink were more likely to choose bottled water, an effect greater for those who had no aesthetic complaints (*p* ≤ 0.001), while those with aesthetic complaints were more likely to choose bottled water if they believed the water was safe (*p* < 0.001). Respondents who treated their water and did not use a community supply were more likely to choose bottled water (*p* < 0.001), while those who did not treat their water were more likely to choose bottled water regardless of whether a community supply was used (*p* < 0.001). A similar pattern of risk factors was associated with a decreased likelihood of consuming tap water daily; however, the use of a community water supply was not significant. Understanding the factors involved in drinking water choices could inform public health education efforts regarding water management in rural areas.

## 1. Introduction

According to a recent Canadian survey [1], 20% of Saskatchewan residents reported choosing bottled water as their primary source of drinking water. Choices around drinking water consumption are governed by a complex set of factors relating to sensory perception, risk perception, and economic, psychological and social factors, including media reports and marketing messages [2]. Additionally, accessibility and cost of bottled water are important factors, especially in rural and remote areas [3]. Several studies have investigated a variety of risk factors associated with aspects of drinking water choices in North America, but few have considered the drinking water choices made by rural residents and we are not aware of any that have exclusively investigated drinking water choices of residents in rural areas of Canada. Previous studies have examined the influence of risk factors on choosing to drink bottled water [4,5,6,7], the risk factors associated with choosing bottled water and using in-home treatment of tap water [8,9], and risk factors associated with choosing tap water, filtered tap water, or bottled water [10].

Perceptions of water quality and risk are important factors in the choice to drink bottled water [2,4,6,10]. Aesthetic qualities of water, particularly taste and odor, also appear to be associated with the choice to drink bottled water [2,6,10,11]. Choosing bottled water has also been associated with age [4,9,10], gender [4,10] , and income [9,10]. Though not examined in many studies, the household’s water source could play a role in the choice to drink bottled water [4,12], and regional differences have also been found [4,9].

Many Canadian studies of drinking water consumption patterns have taken place in urban settings where water quality is routinely monitored, but in rural areas, residents may use a range of tap water sources, including private supplies for which the owner has sole responsibility for monitoring. These supplies can come from surface or ground water sources of variable quality, and they can be impacted by local land use activities [13]. We hypothesized that types of water sources, water quality, and risk perception could be important factors influencing drinking water choices in rural Saskatchewan. The goal of this study was to gain a better understanding of how water sources and water quality and risk perception might influence choices around drinking water in rural Saskatchewan. Our primary objectives were to examine risk factors associated with the choices to consume tap water and bottled water in rural Saskatchewan. We also examined the factors associated with the choice to treat tap water using equipment in the home. 

## 2. Materials and Methods

### 2.1. Design

An anonymous postal questionnaire was administered to 7,500 rural households in six geographic regions of Saskatchewan in the fall of 2011. The questionnaire was distributed through Canada Post’s Unaddressed AdMail service, which provides delivery of bulk mail without specific addresses to houses and farms within a given postal code. Target postal codes were selected by using Canada Post Householder Counts in conjunction with postal code geography files [14]. A geographic information system (ArcMAP, ESRI, Redlands, CA, USA) was used to calculate the smallest radius around a central point selected for each region which would include the centroids of enough postal codes to encompass 1,250 eligible households. To ensure that the questionnaire would be distributed primarily to rural households, postal codes that did not include any farms were excluded, and where postal codes contained more than 200 houses, the survey was sent only to farms within that postal code. 

Questionnaires were sent to 1,250 households from between nine and 12 postal codes (median = 10) in each of the six regions for a total of 60 postal codes. The resultant data included a multistage, hierarchical sample of respondents from households within postal codes selected from within each geographic region. As a result of this distribution process, the questionnaire was delivered to a sample of residents from 24% of the rural municipalities within Saskatchewan.

The four page survey consisted of questions about household water sources, perceptions of quality and health risks from drinking water, consumption of tap water and bottled water, home treatment of tap water, and demographics. The questionnaire was modified from one used in a pilot study in 2010. We requested that the questionnaire be filled out by one member of the household who was over the age of 18, and returned in a pre-addressed, postage-paid envelope. Distribution of reminders or follow up to households that did not respond was not practical given that the questionnaires were not addressed to specific households. Ethics approval was obtained from the University of Saskatchewan Behavioral Research Ethics Board. 

### 2.2. Outcomes and Potential Risk Factors of Interest

The primary outcomes of interest were whether respondents reported primarily drinking bottled water and whether they consumed their household’s tap water daily. Of secondary interest was the choice to treat the household tap water in some way. 

**Figure 1:**
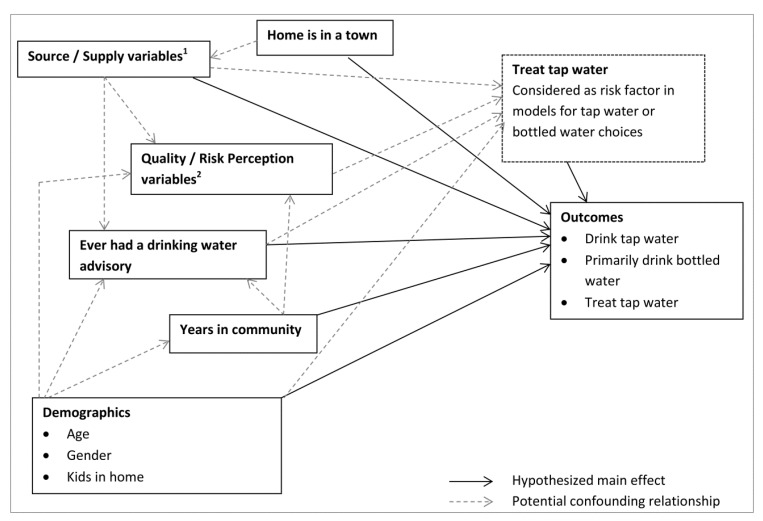
Generalized causal diagram used to direct model development for each of the outcomes related to water consumption and treatment choices (potential interactions not diagrammed for simplicity).

Notes: ^**1**^ Source/supply variables include use of a community (treated) water supply, private water supply, ground water source, or surface water source (none of which are considered mutually exclusive); **^2^** Quality/risk perception variables include having any aesthetic complaint about the tap water, perception that water is not safe to drink, fear water will become contaminated, and perception that tap water has made anyone ill (similarly, none are mutually exclusive).

A causal diagram (Figure 1) was constructed to help guide the process of model development. Primarily choosing bottled water was recorded as a dichotomous variable based on a question about consumption of purchased bottled water in the home with three possible responses. If respondents chose “yes, it is the primary drinking water source” they were classified as primarily bottled water users, whereas those who chose “no” or “yes we drink it sometimes” they were classified as not using primarily bottled water. Daily consumption of tap water was also a dichotomous variable. Treating the tap water was evaluated as a dichotomous outcome based on the response to a question asking if the respondent had any equipment in their home to make the tap water better or safer to drink. Because the use of in-home treatment devices has been examined as a predictor of water consumption patterns in other studies [9,12,15], the use of water treatment in the home was also assessed as a risk factor in the models for bottled and tap water choices. 

Risk factors examined included variables related to household tap water sources: use of a community managed water supply, use of a private water supply, and whether the water source is ground water or surface water. In rural areas, households sometimes use more than one water source; therefore, community and private supplies were not mutually exclusive, nor were ground and surface water sources, so each of these variables was analyzed separately. 

Risk factors related to water quality and risk were also evaluated, including reporting any aesthetic complaint, the perception that tap water was not safe to drink, the fear that the water supply will become contaminated, and the perception that the tap water had made anyone ill. Reporting any aesthetic complaint was a dichotomous variable, recoded from a question for which respondents could select any number of choices from a list of complaints about their tap water. If any of odor, bad taste, discoloration or cloudiness were selected, the respondent was considered as having any aesthetic complaint about their tap water. The perception that the water was not safe to drink, fear that water would become contaminated, and the perception that the tap water had made someone ill were dichotomous variables and were based on questions for which yes or no responses could be given. 

Whether or not the household had ever experienced a drinking water advisory was analyzed as a dichotomous risk factor. Respondents reported whether or not an advisory had ever been experienced, but not reasons for the advisories or the time frame within which past advisories were experienced. The number of years residing in the current community, age, gender, and whether there are children in the household were also analyzed as risk factors. Six age categories were recorded on the questionnaire (18–24, 25–34, 35–44, 45–54, 55–64 and ≥65 years); however, due to low numbers of responses in the three youngest age groups, these were collapsed into a single category, so that only four age categories were used in the analysis (*i.e.*, 18–44, 45–54, 55–64 and ≥65 years). Four possible categories for the number of years residing in the community (0–5, 6–10, 11–20 and 21 or more years) were also collapsed into two categories (≤10 years, >10 years) for analysis. 

### 2.3. Statistical Analysis

Each of the outcomes was modeled using a generalized linear mixed model, specifying a binomial distribution and logit link function. Random intercepts were included in all models for both postal code and geographic region to account for any clustering arising from the hierarchical structure of the data. 

Models were built for each outcome by first screening each risk factor individually where any risk factor with *p* < 0.2 was retained for consideration when building the final model. Manual backwards selection was used to build the final main-effects model, retaining only risk factors with *p* < 0.05. All risk factors dropped from the main effects model were then assessed for confounding based on whether its inclusion in the model led to a change greater than 10% in the regression coefficients for other risk factors. Biologically plausible two-way interactions between risk factors retained in the final model were assessed at a 0.05 level of significance; in the case of categorical variables, a type 3 likelihood ratio test was used to determine if the interaction was significant. 

Models were built in Stata (StataCorp LP, College Station, TX, USA) with the xtmelogit command using a Laplacian approximation for efficiency. Using the risk factors identified in the model building process, the final model parameters were estimated with gllamm, using adaptive quadrature with 12 integration points. Estimates of the random effects and predicted probabilities for each model were produced using gllapred. Population averaged probabilities were estimated using the gllapred marginal function [16].

The proportion of the variance accounted for by postal code and region were examined for each of the outcomes, using an approximation of the variance partition coefficient for the binomial outcome based on the latent response variable model [17]. 

Values were missing for all outcomes and risk factors from at least one survey; any observations that were missing values for any of the risk factors or outcome for a given model were excluded from analyses including that variable. Therefore, the final number of observations used in each model varies and was reported for each model. Model assumptions were examined by evaluating the distribution of the residuals at the postal code and geographic region levels using Q-Q plots. The potential for outliers and influential data points was also investigated by plotting the standardized residuals at each level. 

## 3. Results

### 3.1. Descriptive Statistics

Of the 7,500 questionnaires sent out, 2,074 were returned. Seven were excluded because the postal code identifier had been removed by the respondent, one was excluded because it was blank, and one was excluded for being returned after the cut-off date for responses. As a result, 2,065 responses were used in the analyses, an effective response rate of 27.5%.

The median number of responses for each postal code was 44 (range 2–108) and the median number of responses per region was 353 (range 327–368). Frequencies were calculated for each outcome and risk factor (Table 1). 

**Table 1:** Number of complete and missing responses and the proportion of respondents at each level for the outcomes modeled and risk factors evaluated.

Variable		Complete	Missing	Response	Frequency	
		*n*	*n*		*n*	% ^1^
*Outcomes*						
	Primarily Drink Bottled water	2,030	35	Yes	626	30.8
				No	1,404	69.2
	Drink Tap Water Daily	2,013	52	Yes	1,223	60.8
				No	790	39.2
	Treat tap water in-home ^2^	2,003	62	Yes	953	47.6
				No	1,050	52.4
*Risk Factors*						
	Private water supply	2,059	6	Yes	1,249	60.7
				No	810	39.3
	Community treated water supply	2,059	6	Yes	640	31.1
				No	1,419	68.9
	Ground water source	1,857	208	Yes	1,349	72.6
				No	508	27.4
	Surface water source	1,856	209	Yes	613	33.0
				No	1,243	67.0
	Any aesthetic complaint about tap water	1,984	81	Yes	501	25.3
				No	1,483	74.8
	Believe tap water not safe to drink	1,984	81	Yes	235	11.8
				No	1,749	88.2
	Fear of contamination of water supply	1,988	77	Yes	706	35.5
				No	1,282	64.5
	Anyone ever been ill from tap water	1,784	281	Yes	57	3.2
				No	1,727	96.8
	Ever had water advisory	1,981	84	Yes	485	24.5
				No	1,496	75.5
	Number of years in community	2,046	19	≤10 years ^3^	403	19.7
				>10 years	1,643	80.3
	Home is in a town	2,047	18	Yes	525	25.7
				No	1,522	74.4
	Gender	2,005	60	Female ^3^	1,053	52.5
				Male	952	47.5
	Age	2,050	15	18–44 years ^3^	317	15.5
				45–54 years	446	21.8
				55–64 years	614	30.0
				≥65 years	673	32.8
	Children in the home	1,932	133	Yes	437	22.6
				No	1,495	77.4

Notes: ^**1**^ Proportion of complete observations; **^2^** Used as a risk factor in models for choosing tap water and choosing bottled water; **^3^** Reference category.

To assess the representativeness of our sample, the distribution of age categories and gender in our sample were compared to data from the Canada 2011 Census of Population [18] for the Census Subdivisions corresponding to the rural municipalities included in our survey regions (Table 2).

**Table 2:** Comparison of frequency of key demographic variables in the survey sample population and the Statistics Canada 2011 Census of Population for the rural Census Subdivisions included within the survey regions.

Category	Survey Respondents ^1^		2011 Census of Population (%)
	*n*	(%)	
Female	1,053	52.5	47.3
Male	952	47.5	52.7
18–44 years	317	15.5	35.7
45–54 years	446	21.8	23.2
55–65 years	614	30.0	21.7
≥65 years	673	32.8	19.3

Note: ^**1**^ Total number of respondents: 2005 for gender and 2050 for age.

### 3.2. Choosing Primarily Bottled Water

With respect to drinking water preferences, 30.8% of respondents reported primarily consuming bottled water (Table 1). Of the respondents with a private water supply, 30.7% (376/1,224) reported primarily choosing bottled water, while 28.1% (178/634) of those using a community water supply reported consuming primarily bottled water. Use of other types of water supply were less common; 32.5% (39/120) of those who used a public water station (*i.e.*, a community-maintained, publically available fill station) and 39.5% (47/119) of those whose water was delivered by truck reported primarily using bottled water. 

**Figure 2:**
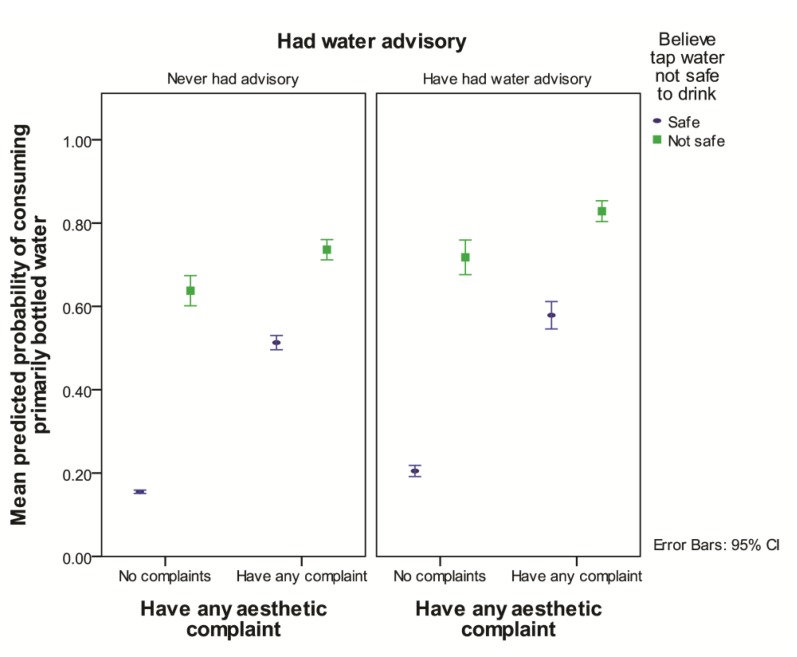
Predicted probability of consuming primarily bottled water by presence of aesthetic complaint and the belief that tap water is not safe to drink, separated by whether or not household had a water advisory in the past averaged over all length of time residing in area, whether a community water supply is used, and in-home treatment of tap water.

After accounting for other significant risk factors, reporting a water advisory increased the likelihood of choosing primarily bottled water (OR = 1.7, *p* < 0.001) compared to not reporting an advisory (Table 3, Figure 2). Of the respondents who reported ever having an advisory, 16.3% (79/485) of the respondents also reported having a current water advisory for their household. Of those that reported current advisories, 60.0% (42/70) reported drinking primarily bottled water and 34.8% (24/69) reported drinking their tap water daily. 

Those who agreed that their tap water was not safe to drink were more likely to consume primarily bottled water than those who did not agree, but the effect of concern about unsafe tap water was greater for those who did not report an aesthetic complaints about the tap water (OR = 8.5, *p* < 0.001) than for those who did (OR = 2.3, *p* = 0.001) (Table 3, Figure 2). Compared to not reporting an aesthetic complaint, reporting any aesthetic complaint about tap water increased the likelihood of primarily consuming bottled water 6.1 times, but only for those who believed the tap water was safe (*p* < 0.001) (Table 3, Figure 2). 

**Figure 3:**
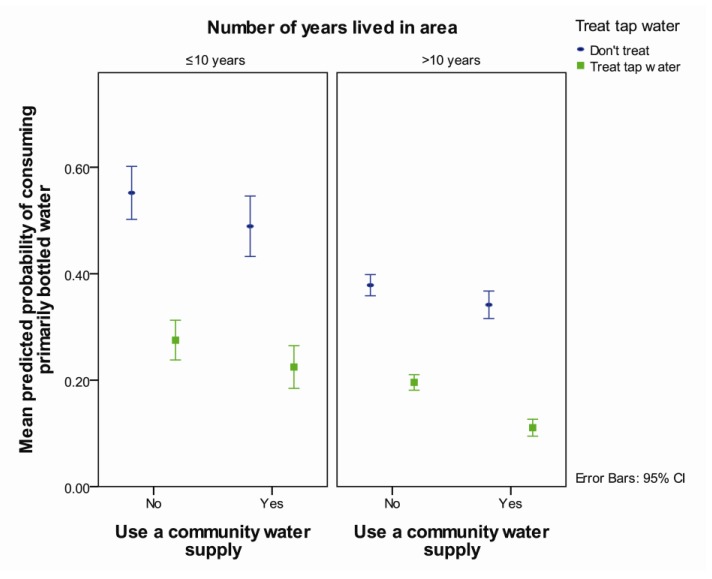
Predicted probability of consuming primarily bottled water by use of a community water supply and in-home treatment of tap water separated by length of residence in the area averaged over reported aesthetic complaints, agreement that tap water is not safe to drink and whether the household experienced a water advisory.

Those who lived in an area for ≤10 years were more likely to consume primarily bottled water than those who had not lived there as long (OR = 1.5, *p* = 0.01) (Table 3, Figure 3). Respondents who treated their tap water in their home and didn’t use a community water supply were more likely to primarily consume bottled water compared those who treated their tap water and used a community supply (OR = 2.5, *p* < 0.001) (Table 3, Figure 3). Compared to those who treated their tap water, respondents that did not treat their tap water were more likely to primarily consume bottled water, but the extent of the increase was greater for those who used a community water supply (OR = 4.6, *p* < 0.001) than for those who did not (OR = 2.5, *p* < 0.001) (Table 3, Figure 3).

The proportion of variance explained by postal code (4.3%) was greater than that explained by region (0.3%) in the final multivariable model for primarily choosing bottled water. This represents a 40% improvement in the variance explained by postal code compared to the random effects of the null model, in which postal code accounted for 7.1% of the variance and region accounted for 0.4%. 

**Table 3:** Risk factors associated with the primary consumption of bottled water in the final multivariable model.

Risk Factor				OR	95% CI		*p*
Ever had water advisory				1.7	1.3	2.4	<0.001
Lived in area for > 10 years				Ref ^1^			
Lived in area ≤; 10 years				1.5	1.1	2.0	0.01
Believe water not safe × have any aesthetic complaint							<0.001 ^2^
	Believe tap water is not safe to drink, compared believing it is safe, for those who have any aesthetic complaint			2.3	1.4	3.8	0.001
	Believe tap water is not safe to drink, compared to believing it is safe, for those who have no aesthetic complaints			8.5	5.2	13.9	<0.001
	Have any aesthetic complaint about tap water compared to not having a complaint, for those who believe their tap water is not safe			1.7	0.9	3.2	0.13
	Have any aesthetic complaint about tap water compared to not having a complaint, for those who believe their tap water is safe			6.1	4.6	8.0	<0.001
Use a community water supply × treat tap water							0.03 ^2^
		Not using a community water supply compared to using a community supply, for those who treat the tap water		2.5	1.5	4.0	<0.001
		Not using a community water supply compared to using a community supply, for those who do not treat their tap water		1.3	0.9	1.9	0.10
		Not treating tap water compared to treating tap water, for those who use a community water supply		4.6	2.9	7.3	<0.001
		Not treating tap water compared to treating tap water, for those who do not use a community water supply		2.5	1.9	3.3	<0.001
**Variances of Random Effects**				**Variance**	**SE**		
		Postal code		0.147	0.086		
		Region		0.010	0.030		
Number of observations = 1,844							

Notes: ^**1**^ Reference category; **^2^** Overall p-value for interaction based on type 3 likelihood ratio test.

### 3.3. Consuming Tap Water Daily

Most people (74.6%, (1,518/2,036)) reported consuming their tap water at least some of the time and 60.8% reported drinking tap water on a daily basis (Table 1). Of the respondents who used a private water supply, 63% (762/1216) reported consuming tap water daily. Daily tap water consumption was also reported by 61% (380/628) of respondents who used a community supply, 54% (63/117) of those who used truck-delivered water, and 55% (54/117) of those who used a public water station. 

After accounting for other significant risk factors, reporting a water advisory decreased the likelihood of consuming tap water daily compared to not reporting an advisory (OR = 0.7, *p* = 0.004) (Table 4). Those who lived in an area >10 years were 1.6 times more likely to consume tap water daily (*p* < 0.001) than those who lived in an area for a shorter time (Table 4). 

**Table 4:** Risk factors included in final multivariable model for daily consumption of tap water.

Risk Factor			OR	95% CI		*p*
Ever had water advisory			0.7	0.5	0.9	0.004
Lived in area < 10 years			Ref. ^1^			
Lived in area > 10 years			1.6	1.2	2.1	0.001
Believe tap water not safe × any aesthetic complaint						0.001 ^2^
	Believe tap water not safe to drink compared to believing it is safe, for those with any aesthetic complaint		0.4	0.2	0.7	0.001
	Believe that tap water not safe to drink compared to believing it is safe, for those with no aesthetic complaints		0.1	0.1	0.2	<0.001
	Have any aesthetic complaint compared to not having any aesthetic complaint, for those who believe the tap water is not safe		0.5	0.2	1.1	0.09
	Have any aesthetic complaint compared to not having any aesthetic complaint, for those who believe the tap water is safe		0.1	0.1	0.2	<0.001
Have any aesthetic complaint × treat tap water						0.006 ^2^
	Have any aesthetic complaint compared to not having any aesthetic complaint, for those who treat their tap water		0.2	0.2	0.3	<0.001
	Have any aesthetic complaint compared to not having any aesthetic complaint, for those who do not treat their tap water		0.1	0.1	0.2	<0.001
	Treat tap water compared to not treating tap water, for those with any aesthetic complaint		3.7	2.4	5.7	<0.001
	Treat tap water compared to not treating tap water, for those with no aesthetic complaints		1.8	1.4	2.4	<0.001
**Variances of Random Effects**			**Variance**	**SE**		
		Postal code	0.106	0.064		
		Region	2.4 × 10^−15^	6.4 × 10^−8^		
Number of observations = 1,830						

Notes: ^**1**^ Reference category; **^2^** Overall *p*-value for interaction based on type 3 likelihood ratio test.

**Figure 4:**
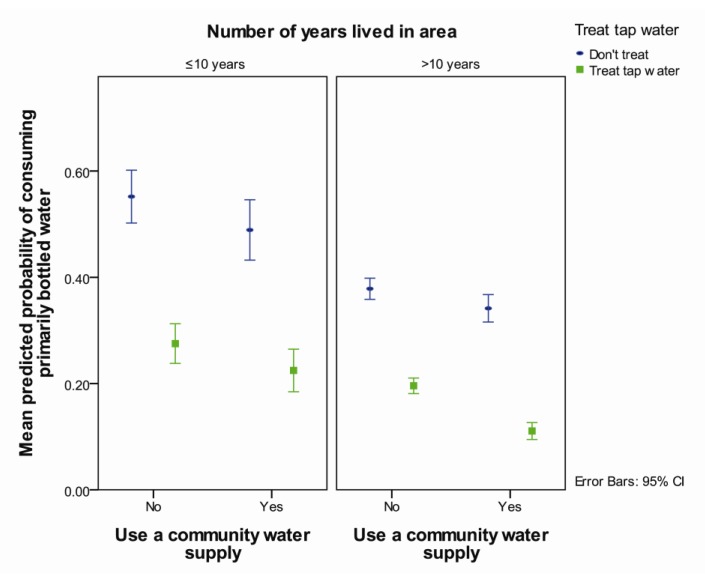
Predicted probability of consuming tap water daily by presence of aesthetic complaint and the belief that tap water is not safe to drink, separated by in-home treatment of tap water averaged over all whether household had experienced a water advisory and length of time resided in area.

Those who did not think that their tap water was safe to drink were less likely to consume tap water daily than those who did, but the magnitude of the effect was slightly smaller for those who also reported aesthetic complaints (OR = 0.4, *p* < 0.001) than for those who did not (OR = 0.1, *p* < 0.001) (Table 4, Figure 4). When compared to those who believed their tap water was safe and had no aesthetic complaints, respondents who though their tap water was safe, but had at least one aesthetic complaint were 10 times less likely to consume their tap water daily (*p* < 0.001) (Table 4, Figure 4).

Reporting at least one aesthetic complaint also decreased the likelihood of consuming tap water daily for all respondents, with the effect of an aesthetic effect being greater for those who did not treat their tap water (OR = 0.1, *p* < 0.001) than for those who did (OR = 0.2, *p* < 0.001) (Table 4, Figure 4). Those who treated their tap water in some way were more likely to consume tap water daily compared to those who did not, but the importance of treatment was greater for those who also reported an aesthetic complaint (OR = 3.7, *p* < 0.001) than for those who had no complaints (OR = 1.8, *p* < 0.001) (Table 4, Figure 4).

The proportion of the variance in the final multivariable model for consuming tap water daily explained by postal code was 3.1%, while a negligible proportion was explained by region of the province (<0.001%). This represents a slight improvement compared to the random effects in the null model, in which postal code accounted for 4.9% of the variance and region accounted for 0.4%. The main effects in this model explained nearly 100% of the already small variation between regions of the province, and 38% of the variation between postal codes.

### 3.4. In-home Treatment of Tap Water

Of the respondents, 47.6% reported that they treat their household tap water in some way (Table 1). Of the respondents using a private water supply, 52.4% (637/1,215) reported treating their tap water, while 42.5% (264/621) of those who used a community water source reported treating their tap water in the home. Of those who used a surface water source, 41.6% (248/596) treated their water, while 50.7% (666/1,314) of those who used a ground water source reported treating their tap water. Of the respondents who indicated which type of treatment they used, 58.7% (501/853) used a water softener, including 68.2% (416/610) of those using a ground water source and 34.1 (76/233) of those using a surface water source. Of all respondents using water softeners, 72.1% (361/501) also indicated that some other form of water treatment (e.g., reverse osmosis, jug filter, ultraviolet, and distillation) was used. 

**Figure 5:**
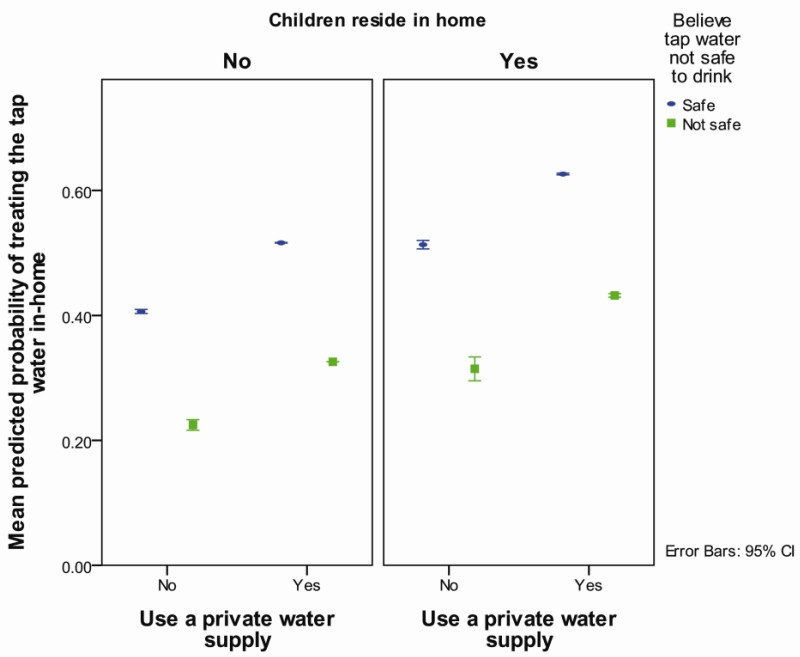
Predicted probability of treating tap water in-home by use of a private water supply and the belief that tap water is not safe to drink, separated by whether or not children reside in household, adjusted for whether the home was in a town and whether a community water supply was used.

After accounting for other risk factors, those who used a private supply were more likely (OR = 2.1, *p* < 0.001) to treat their household water compared to those who did not use a private supply (Table 5, Figure 5). Those who reported their tap water was not safe were half as likely to treat their tap water (*p* < 0.001) (Table 5, Figure 5) than those who did not. Having children under 18 residing in the home increased the likelihood of treating the tap water (OR = 1.6, *p* < 0.001) compared to not having children in the home (Table 5, Figure 5). Estimates were adjusted to minimize potential confounding by whether the home was in a town, and whether or not a community water supply was used. 

The random effects explained only a small proportion in the variation between postal code (2.0%) and region (1.1%) in the final multivariable model with little change from the proportions in the null model (2% and 1.5% respectively). This suggested there were few differences among postal codes and regions regarding the decision to treat water.

**Table 5:** Risk factors included in final multivariable model for in-home treatment of tap water.

Risk Factor	OR	95% CI		*p*
Use a private water supply	2.1	1.5	3.0	<0.001
Believe tap water not safe	0.5	0.3	0.6	<0.001
Children reside in home	1.6	1.2	1.9	<0.001
Home is in a town	1.2	0.8	1.7	0.31
Use a community water supply	1.3	0.9	1.8	0.25
**Variances of Random Effects**	**Variance**	**SE**		
Postal code	0.067	0.044		
Region	0.038	0.035		
Number of observations	1,796			

The risk factors identified for consuming primarily bottled water, consuming tap water daily and choosing to treat tap water were summarized in Table 6.

**Table 6:** Summary of the analyses for consuming primarily bottled water, consuming tap water daily and choosing to treat tap water, including the number of observations used and the risk factors associated with the final model for each outcome (effect modifications italicized).

Outcome	*n*	Factors that Increase Likelihood of Outcome	Factors that Decrease Likelihood of Outcome
Primarily choose bottled water	1,711	History of water advisory *Believe tap water is not safe to drink—magnitude of effect greater for those that have no aesthetic complaints* *Have any aesthetic complaints about tap water, only for those who believe their tap water is safe*	Lived in area longer than 10 years *Use a community water supply, only for those who treat tap water* *Treat the tap water, with the magnitude of effect larger for those who use a community water supply*
Consume tap water daily	1,818	Lived in area longer than 10 years *Treat tap water—magnitude of effect larger for those that also have aesthetic complaint about tap water*	History of water advisory *Believe tap water not safe to drink—magnitude of effect greater for those with no aesthetic complaint* *Have any aesthetic complaint, only for those who belief the tap water is safe to drink* *Have any aesthetic complaint—magnitude greater if tap water not treated*
Treat tap water ^1^	1,796	Use a private water supply Children reside in the home	Believe tap water not safe to drink

Note: ^1^ Adjusted for whether home is in a town and whether a community water supply is used.

## 4. Discussion

In the present study, we investigated risk factors associated with water consumption choices among rural Saskatchewan residents, using quantitative analyses to examine the combined influence of several risk factors and account for the potential for clustering by community. The outcomes under investigation were not mutually exclusive, and the risk factors underlying the choice to primarily consume bottled water and to consume tap water daily were similar, although as expected their effects were opposite for these two outcomes. The models for primarily choosing bottled water and for daily consumption of tap water both included length of residence in the area, having had a water advisory, and an interaction between the belief that the tap water is not safe to drink and having any aesthetic complaint about the tap water. The risk factors for in-home treatment of the tap water included the use of a private water source, belief the water was not safe to drink, and whether children resided in the home.

### 4.1. Consuming Primarily Bottled Water

Just over 30% of our respondents reported using primarily bottled water, with little difference between those who used a community water supply and those who used a private water supply. A recent national Canadian survey [1] reported that 20% of all Saskatchewan respondents reported consuming primarily bottled water, and that 19% of respondents using a municipal water supply and 39% of respondents using non-municipal supplies reported using primarily bottled water. However, 93% of the Saskatchewan respondents to the Statistics Canada survey [1] reported using a municipal water supply, compared to just 31% of respondents in our rural study population.

We did not attempt to quantify the proportion of bottled water consumed that would define a respondent as choosing primarily bottled water; whereas, some water consumption studies have set a threshold of greater than 75% bottled water use (e.g., [8,9]). Other Canadian studies had rates of primary bottled water use ranging from 22% [1] to 35% [19]. A recent national US study reported that just 13% of respondents reported using bottled water [4]. 

Among rural Saskatchewan residents, the choice to consume primarily bottled water at home appears to be mediated by a number of related factors. An interaction was identified between the belief that the tap water is not safe to drink and whether the respondent had any aesthetic complaints about the tap water. Having any aesthetic complaint increased the likelihood of choosing primarily bottled water, but only for respondents who felt their tap water was safe. Those who felt their tap water was not safe to drink were consistently more likely to choose bottled water than those who felt it was safe regardless of aesthetic concerns. 

Although this interaction between these risk factors is unique to the present study, our findings build on previous studies which reported that bottled water use was related to aesthetic complaints [6,10,11,19] and perception of health risks from tap water [4,5,6,7,10,19,20].

Another interaction was identified between use of a community water source and whether respondents use some sort of in home treatment for their tap water. People who used a community water supply compared to some other supply were less likely to choose bottled water, but this was only true for those who treated their tap water. Respondents that treated their tap water were also less likely to consume primarily bottled water than those who did not, but the difference was greater for those that used a community water supply. Home treatment was identified as a factor that reduces the likelihood of consuming primarily bottled water in previous studies [8,9]. Its interaction with the use of a community water supply might reflect the use of home treatment devices to remove chlorine taste from tap water [9]. 

Respondents who reported having a water advisory were more likely to primarily consume bottled water. To our knowledge this has not been previously investigated as a risk factor. Drinking water advisories in Saskatchewan are issued for a variety of reasons. For larger distribution systems (flow rate > 18,000 L/day flow) precautionary advisories were most commonly issued in the year prior to our survey for operational reasons such as depressurization of the system, line breaks, planned maintenance or high turbidity levels which could compromise treatment, while in smaller systems precautionary advisories more often resulted from positive bacteriological testing. In both types of systems, emergency boil water orders were most often due to finding coliform bacteria in the water [21]. It is possible that having experienced an advisory could introduce doubts about the safety of the household water. Because those respondents reporting an advisory included some respondents that were currently under an advisory, the possibility that being under a current advisory was driving the consumption of bottled water was considered. However, relatively few respondents (16%) who reported ever having a water advisory also reported a current water advisory for their household. Furthermore, of the respondents with a current advisory, over a third reported drinking their tap water daily. Although this suggests that respondents might drink their tap water despite being under an advisory, it is also possible that respondents reported their typical daily beverage choices as opposed to the choices made specifically during an advisory situation. Given the importance of experiences with water advisories in choices made around drinking water the impact of water advisories on drinking water habits should be studied in more depth. 

Respondents that had lived in the area for longer than 10 years were less likely to choose bottled water. Familiarity with the tap water has been identified as an important factor in perception of water quality [22,23], and this result suggests that familiarity also reduces the likelihood that alternatives to tap water will be sought. 

The role of demographic variables such as age and gender has been inconsistent in previous studies [3]. Although age was unconditionally associated with primarily choosing bottled water in the present study, age was not included in our final model for primarily consuming bottled water. Some studies have reported that age as a significant predictor of bottled water usage [4,8,9] with the consumption of bottled water declining above age 30 in at least two studies. The population of respondents to our questionnaire was skewed toward older age groups which could have reduced the power of our study to detect differences between older and younger age groups. However, it is also possible that the effects of age are mediated through other risk factors included in our model.

The role of gender is less clear. In some previous analyses that examined the effects of gender in isolation, being female was associated with increased consumption of bottled water [6,10]. However, using multivariable analysis, Dupont [10] reported that males with children were more likely to be bottled water users than males without children, while Hu [4] reported that females had increased odds of drinking bottled water. However, gender was not a significant risk factor in another study [5] or in the present study. It is possible the effect of gender depends on other factors in the population under study or its effects are partly mediated through other risk factors such as perceptions of quality and risk. 

### 4.2. Consuming Tap Water Daily

More than 60% of our respondents reported drinking their tap water on a daily basis. We did not clarify if these respondents were primarily consuming tap water, but just 3% (40/1,213) of the respondents in this group also indicated that they primarily consume bottled water. Consequently, daily consumption of tap water was the only measure we had available to classify respondents as regular users of tap water. Considering that some respondents may not typically consume water on a daily basis, we could have underestimated the number of respondents who primarily consume tap water. In a national Canadian survey [1], 76% of Saskatchewan residents reported consuming primarily tap water. In the same survey, 78% of residents on a municipal supply reported drinking tap water, while only 49% of those with private water supplies chose primarily tap water. Among our respondents the proportion of respondents that reported drinking tap water daily was similar among users of private and community supplies. 

The groups of risk factors included in the final model for choosing to drink tap water daily were similar, though not identical, to those for choosing primarily bottled water, but with opposite effects. Survey participants that reported water advisories were less likely to report daily tap water consumption, suggesting that this experience might reduce their confidence in the safety of tap water. Living in an area for longer than 10 years was perhaps a measure of familiarity with the water, and increased the likelihood of consuming tap water daily. 

Reporting any aesthetic complaint decreased the likelihood of daily tap water consumption, but its effect was modified by both whether residents believed the tap water was safe, and whether residents treated their tap water; these interactions appeared to be independent of each other. 

Reporting an aesthetic complaint made it less likely that tap water would be consumed regularly, but only when the tap water was considered safe. The belief that the tap water was not safe to drink made it less likely that tap water would be chosen regardless of the presence of any aesthetic complaints. This interaction was the inverse of a similar interaction found in the model for choosing to consume primarily bottled water.

The effect of reporting an aesthetic complaint on the likelihood of consuming the tap water was somewhat mitigated by treating the tap water, and treating the water had a greater effect on the likelihood of consuming tap water when an aesthetic complaint was reported. This interaction was important to the likelihood of choosing to consume tap water daily; whereas, having an aesthetic complaint was not an important risk factor for the decision to treat the water on its own. It appears that aesthetic qualities are important to the decision to treat only in to the context of whether the tap water is consumed regularly. 

The choice to treat tap water was evaluated as a risk factor for the consumption of tap water even though the direction of the causal relationship between treating tap water and drinking tap water is not clear. For instance, the tap water might be chosen because of the perception that treatment has made it more safe or palatable, or the decision to treat might be made if tap water is the only viable option for drinking water and it is perceived to not be safe or palatable unless treated. 

Few previous studies have examined the risk factors associated with primarily choosing to drink tap water. Dupont *et al.* [10] used analysis of variance to examine factors associated with the proportion of tap water consumed relative to filtered and bottled water in Canada, and found that the degree of concern about health risks from tap water was inversely related to the proportion of tap water consumed, as was the presence of various aesthetic concerns. This was similar to our results and underscores the importance of perception of quality and risk in making choices about drinking water. 

Given the similarity between the models for primarily consuming bottled water and regularly consuming tap water, it might be reasonable to assume that similar factors, acting in opposite directions, play a role in each choice. However, there were some differences in the risk factors for each choice, and it has been hypothesized that choosing bottled water is not necessarily an alternative to choosing tap water, but may instead be considered an alternative to other pre-packaged beverages such as soda and juice [3,24]. The relative importance of bottled water as an alternative to tap water compared to other beverages requires further investigation. 

### 4.3. Treating Tap Water

We also investigated the risk factors associated with the decision to treat the household tap water. Use of in-home water treatment devices has become common [25]. Statistics Canada [1] reported that 50% of Saskatchewan residents indicated that they treat their tap water with a purifier, filter, or by boiling prior to consumption. More than 47% of our respondents indicated that they used any type of equipment in the home intended to make the tap water “better or safer to drink.” This number includes respondents that used water softeners, which are not recommended for the treatment of drinking water [25]. In other Canadian studies, water softeners were included as treatment devices, and rates of water treatment were similar [8,9]. In the present study, of those who indicated the type of treatment device used, 72% of respondents who used a water softener also used another device intended to treat drinking water. 

Our results indicated that believing the tap water was not safe reduced the likelihood of treating the tap water. This is contradictory to another cross-Canada study that found health concerns increased the likelihood of consuming filtered tap water [10]. However, there could be a substantial difference between having general health concerns about water and the belief that the water is not safe. It is possible that if respondents felt their tap water was unsafe, they had no intention of consuming the water so did not treat it, or did not trust that home treatment devices would make their water safe. 

Use of a private water supply increased the likelihood that water would be treated, a finding opposite to a study in British Columbia that reported fewer private source users than expected treated their tap water in an unconditional analysis [9]. This discrepancy may be related to the high rate of use of water softeners among our private water supply users. A study in Nova Scotia also reported that respondents with private water supplies were less likely to treat their water than those connected to a municipal supply [26]. However, in that study the use of bottled water was considered a type of water treatment making it difficult to directly compare their results with the present study, where bottled water consumption was considered separate from treatment. 

We did not find an association between perception of poor aesthetic quality and the decision to treat the tap water among our respondents. This finding contradicted the study performed in Nova Scotia which found that treating household water was associated with the perception of lower water quality [26]. However, as previously mentioned, the risk factors identified in the Nova Scotia study may differ from ours because we evaluated bottle water consumption separately from other types of treatment. 

The presence of children in the home increased the likelihood that the tap water would be treated. Dupont *et al.* [10] found a similar relationship but only for males, whereas gender was not included in our final model. Our model did include confounding variables, suggesting that the factors leading to water treatment are complex and deserving of further study, especially with respect to clarifying the factors related to treatment intended to make drinking water safer or more palatable compared to addressing the mineral content of the water. 

### 4.4. Limitations

As previously discussed, our models for tap water and treatment were limited by self-reported measures of relative tap water consumption and the goals of treatment. It would have been ideal to be able to model the risk factors for choosing primarily tap water for comparison to choosing primarily bottled water, rather than comparison to drinking their tap water on a daily basis. Previous studies have suggested that water consumption decreases with age [8,9,15], which could make daily tap water consumption an especially poor proxy for choosing primarily tap water in older age groups. Overall, the purposive nature of our regional sampling and a relatively low response rate, especially among younger age groups (Table 2), might limit the generalizability of our findings.

## 5. Conclusions

By surveying residents of rural Saskatchewan in different communities and different regions, we were able to estimate the importance of some factors involved in drinking water choices among respondents who have access to a variety of water supplies. While our study provides information about the relationships between factors related to water supply and water quality and risk perception and bottled water use in rural Saskatchewan, there are likely many other factors that are involved including accessibility, convenience, marketing, social cues, and concerns about environmental waste [3]. We examined risk factors associated with the decision to regularly consume tap water. While these were similar to those involved in influencing the choice to drink bottled water, is has also been suggested that consumers don’t necessarily view bottled water as an alternative to tap water, but to other types of beverages such as soda and juice [3,24]. Further investigation of specific perceptions related to water quality and risk, especially in conjunction with estimates of the relative amounts of bottled water, tap water, and other beverages consumed is needed to better understand the drinking water and beverage choices made by residents of rural Saskatchewan. A better understanding of the factors involved in such decisions, and any regional differences in these factors, are crucial for informing public health efforts regarding the safety, testing and treatment of drinking water, as well as the assessment of health risks related to water consumption in rural areas. 

## References

[1] Statistics Canada, Government of Canada Households and the Environment: Analysis. http://www.statcan.gc.ca/pub/11–526-x/2013001/part-partie1-eng.htm.

[2] Doria M.F., Pidgeon N., Hunter P.R. (2009). Perceptions of drinking water quality and risk and its effect on behaviour: A cross-national study. Sci. Total Environ..

[3] Doria M.F. (2006). Bottled water *vs*. tap water: Understanding consumers’ preferences. J. Water Health.

[4] Hu Z., Morton L.W., Mahler R.L. (2011). Bottled water: United States consumers and their perceptions of water quality. Int. J. Environ. Res. Public. Health.

[5] McSpirit S., Reid C. (2011). Residents’ perceptions of tap water and decisions to purchase bottled water: A survey analysis from the appalachian, big sandy coal mining region of west Virginia. Soc. Nat. Resour..

[6] Saylor A., Prokopy L.S., Amberg S. (2011). What’s wrong with the tap? Examining perceptions of tap water and bottled water at Purdue university. Environ. Manage..

[7] Merkel L., Bicking C., Sekhar D. (2012). Parents’ perceptions of water safety and quality. J. Community Health.

[8] Jones A., Dewey C., Doré K., Majowicz S., McEwen S., Waltner-Toews D. (2006). Drinking water consumption patterns of residents in a Canadian community. J. Water Health.

[9] Jones A.Q., Majowicz S.E., Edge V.L., Thomas M.K., MacDougall L., Fyfe M., Atashband S., Kovacs S.J. (2007). Drinking water consumption patterns in British Columbia: An investigation of associations with demographic factors and acute gastrointestinal illness. Sci. Total Environ..

[10] Dupont D., Adamowicz W.L., Krupnick A. (2010). Differences in water consumption choices in Canada: The role of socio-demographics, experiences, and perceptions of health risks. J. Water Health.

[11] Levallois P., Grondin J., Gingras S. (1999). Evaluation of consumer attitudes on taste and tap water alternatives in Quebec. Water Sci. Technol..

[12] Pintar K.D.M., Waltner-Toews D., Charron D., Pollari F., Fazil A., McEwen S.A., Nesbitt A., Majowicz S. (2009). Water consumption habits of a south-western Ontario community. J. Water Health.

[13] Corkal D., Schutzman W.C., Hilliard C.R. (2004). Rural water safety from the source to the on-farm tap. J. Toxicol. Environ. Health Pt A.

[14] DMTI Platinum Postal Suite Version 2006.4: Saskatchewan Local Delivery Unit Area (LDU) Boundaries. http://datalib.usask.ca.cyber.usask.ca/gis/Data/DMTI_GIS_isonequinox/dmti/postcode/platinum_postal_suite/v2012.3/.

[15] Roche S.M., Jones A.Q., Majowicz S.E., McEwen S.A., Pintar K.D. (2012). Drinking water consumption patterns in Canadian communities (2001–2007). J. Water Health.

[16] Rabe-Hesketh S., Skrondal A. (2008). Multilevel and Longitudinal Modelling, Using Stata.

[17] Browne W.J., Subramanian S.V., Jones K., Goldstein H. (2005). Variance partitioning in multilevel logistic models that exhibit overdispersion. J. Roy. Statist. Soc. Ser. A Stat..

[18] Statistics Canada Census of Canada, 2011: Profile of Census Subdivisions (Public-use Microdata File). http://datacentre2.chass.utoronto.ca.cyber.usask.ca/cgi-bin/census/2011/.

[19] Jones A.Q., Dewey C.E., Doré K., Majowicz S.E., McEwen S.A., David W.-T., Eric M., Carr D.J., Henson S.J. (2006). Public perceptions of drinking water: A postal survey of residents with private water supplies. BMC Public Health.

[20] Auslander B.A., Langlois P.H. (1993). Toronto tap water: Perception of its quality and use of alternatives. Can. J. Public Health.

[21] Saskatchewan Ministry of Environment 2010–2011 State of Drinking Water Quality in Saskatchewan. http://www.saskh2o.ca/PDF/EPB418DrinkingWaterAnnualReport10-11.pdf.

[22] Dietrich A.M. (2006). Aesthetic issues for drinking water. J. Water Health.

[23] Doria M.F. (2010). Factors influencing public perception of drinking water quality. Water Policy.

[24] Jones A.Q., Dewey C.E., Doré K., Majowicz S.E., McEwen S.A., Waltner-Toews D., Henson S.J., Mathews E. (2007). A qualitative exploration of the public perception of municipal drinking water. Water Policy.

[25] Health Canada, Government of Canada Water Treatment Devices for the Removal of Taste, Odour and Chemicals. http://www.hc-sc.gc.ca/ewh-semt/pubs/water-eau/devices-dispositifs-eng.php.

[26] Janmaat J. (2007). A little knowledge: Household water quality investment in the Annapolis valley. Can. J. Agr. Econ..

